# Fulminant mediastinitis after goiter recurrence surgery: a case report

**DOI:** 10.1186/1752-1947-4-364

**Published:** 2010-11-17

**Authors:** Susanne Rein, Martina Mittag-Bonsch

**Affiliations:** 1 University Hospital 'Carl Gustav Carus', Department of Trauma and Reconstructive Surgery, Fetscherstr. 74, 01307 Dresden, Germany; 2 Department of Orthopedics, Trauma, Hand, Visceral and Minimally Invasive Surgery, Hospital of Crailsheim, Gartenstr. 21, 74564 Crailsheim, Germany

## Abstract

**Introduction:**

Necrotizing soft tissue infection is a life-threatening disease characterized by rapid progressive inflammation and necrosis of the subcutaneous and deep fascia with or without involvement of the adjacent muscles.

**Case presentation:**

We report the case of a 62-year-old Caucasian woman with goiter recurrence who underwent a right-sided hemithyroidectomy. Postoperatively, she developed fulminant mediastinitis caused by group A β-hemolytic streptococcus and septic shock. Our patient survived this rare life-threatening complication.

**Conclusions:**

Initial atypical postoperative symptoms, such as personality changes or an unstable circulatory system, should lead a practitioner to consider the possibility of this severe complication and to begin therapy immediately.

## Introduction

Necrotizing soft tissue infection (NSTI) is a life-threatening disease characterized by rapid, progressive inflammation as well as necrosis of the subcutaneous and deep fascia with or without involvement of the adjacent muscles. The prevalence of this disease is such that the average practitioner will see only one or two cases in his or her career [1]. NSTI of the head and neck is thus an extremely rare occurrence in modern medicine [2]. We report a case of NSTI after goiter recurrence surgery.

## Case presentation

A 62-year-old Caucasian woman presented with a six year history of a growing mass in the right side of her neck. Our patient reported that she had had a euthyroid nodular goiter with a compensated autonomous adenoma and suppression of the residual thyroid 21 years previously. At that time, a subtotal thyroid resection of both lobes was performed. The postoperative period was unremarkable.

Clinical examination revealed a right-sided nodular goiter, which moved with swallowing. There was no dyspnea. Laboratory tests showed euthyroid metabolism. Scintigraphy results revealed a non-homogeneous right-sided recurrent goiter with some central cold areas. An ultrasound scan showed a right central richly echogenic nodule surrounded by otherwise poorly echogenic nodular tissue. The volume of the right thyroid lobe was 82 mL, and that of the left thyroid lobe was 3 mL.

Our patient's medical history included metabolic syndrome with second-degree obesity as indicated by a body mass index of 39 kg/m^2^, orally treated diabetes mellitus type 2b, stage II arterial hypertension according to World Health Organization (WHO) criteria, compensated renal insufficiency following a left nephrectomy for a hypernephroid clear-cell carcinoma (p-T2N0M0G1) in 1985 and an uncertain penicillin allergy, although piperacillin was tolerated with no problems during the reported treatment. Cardiac and rhythmogenic problems as well as epilepsy were excluded one year previously because of drop attacks with an undetermined cause. An episode of pyelonephritis was also treated one year previously with antibiotics. In addition, there was no history of any of the following pre-existing diseases and risk factors: sore throat, alcohol abuse, dermatitis or ulcers, *Varicella zoster *infection, or recent invasive treatments, injections or operations. There was no history of muscle or skin injuries, thrombotic tendency or infections contracted from a family member or from personal surroundings. Regular follow-up investigations of the cause of the renal carcinoma showed neither relapse nor metastasis, nor a second cancer.

Surgery was recommended because of the increasing size of the right-sided recurrent nodular goiter with cold areas. A right near-total or complete resection was proposed, depending on the intra-operative findings. After extensive discussion with our patient about the proposed surgery, its possible complications and alternative therapies, she decided to undergo the surgical treatment.

A right hemithyroidectomy with neuromonitoring of the laryngeal recurrent nerve was performed. Our standard pre-operative antiseptic procedure was performed, including triple alcohol disinfection of the operation area over five minutes and a single-use sterile covering of the operation site. Our patient did not receive pre-operative antibiotic prophylaxis because her medical history had revealed no increased infection risk.

The exploration of the left thyroid lobe showed a very small, homogeneous and unremarkable thyroid remnant, which was left in place. A drain was placed in the area of the right thyroid lobe and the surgical procedure was completed without complications after 85 minutes.

Our patient was observed for one night in our intensive care unit, which is standard procedure in our department. A temporary increase in our patient's body temperature to 38.6°C was noted in the late afternoon, but this returned to normal by the evening without further intervention. Our patient was able to return to the visceral surgical ward after an unremarkable night and normal laboratory values on testing.

**Figure 1:**
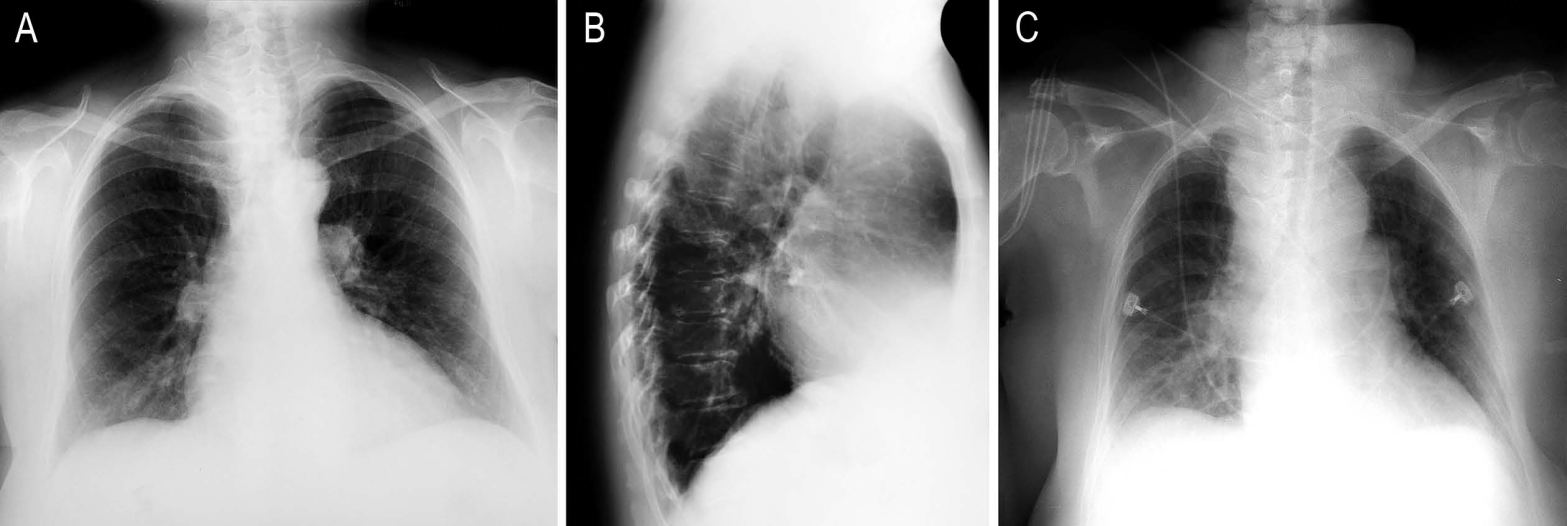
**Pre-operative chest radiographs in two planes with an enlarged cardiac silhouette (A, B)**. The anteroposterior (ap) chest X-ray already shows a discrete mediastinal enlargement and a right basal shadow at 9.30 p.m. on the first postoperative day (C). Note the postoperative drainage in the right thyroid loge.

**Figure 2:**
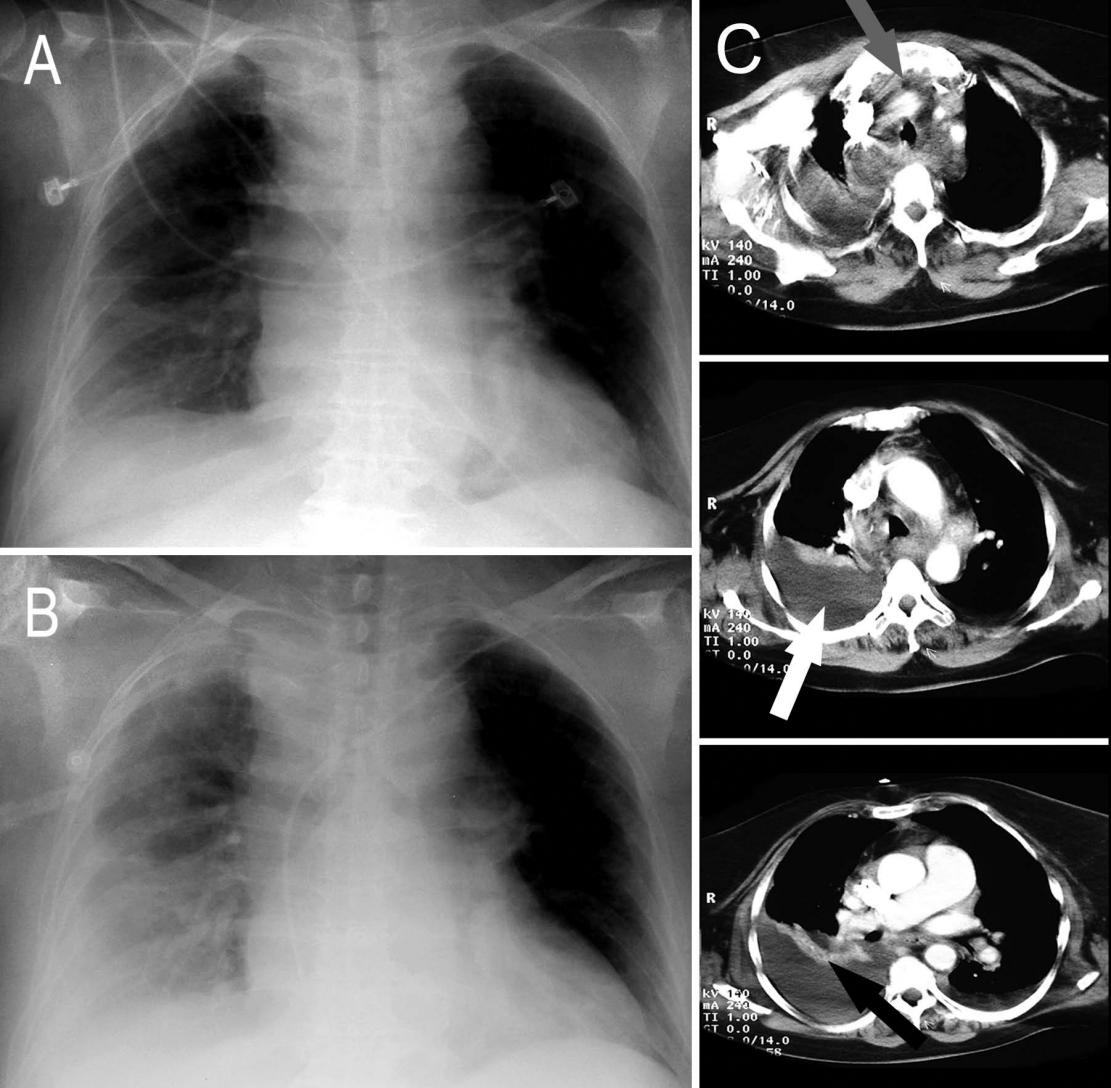
**Anteroposterior chest radiographs at 7.15 a.m. (A) and 1.30 p.m. (B) on the second postoperative day**. A right pleural effusion already existed in the morning (A) and increased in size until 12 p.m. (B). Computed tomography (CT) scans of the thorax (C) performed on the second postoperative day verified a significant increase in fluid volume in the upper mediastinum (upper grey arrow). Furthermore, a large pleural effusion was observed (white arrow in the middle) with atelectasis of the right middle lobe (right black arrow). The subcutaneous air pockets described in cases of necrotizing soft tissue infection (NSTI) in the literature were absent.

Our patient presented with personality changes, dizziness and arterial hypotension, with systolic values between 80 and 100 mmHg on the evening of the first postoperative day, 29 hours after surgery. Her infusion therapy was continued. A short-term increase in our patient's body temperature to 38.5°C was again noted. Her wound appeared normal. There was no pain. She was returned to our intensive care unit on the evening of the first postoperative day due to increasing deterioration of her general condition and the need for careful observation. Arterial and central venous catheters were applied. Radiological diagnostic tests were performed (Figure 1). Radiology (Figure 2), microbiology and laboratory investigations were performed on the morning of the second postoperative day. The wound was still unremarkable. No increase in body temperature was observed. Immediate revision surgery was indicated under suspicion of mediastinitis with an unknown bacterium or NSTI. Intravenous antibiotic therapy, including piperacillin 3 × 4 g/day, gentamicin 160 mg/day and vancomycin 2 × 500 mg/day, was given.

**Figure 3:**
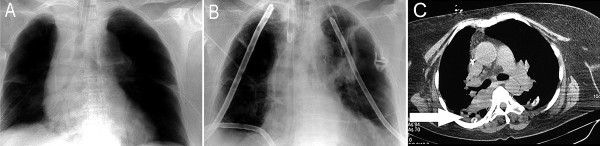
**Anteroposterior chest radiographs from the third postoperative day.** (A) showing the chest tubes in place on both sides after the second revision surgery. B) The anteroposterior chest radiograph from the fourth postoperative day after another surgical revision involving mediastinotomy, pericardiostomy as well as placement of suction lavage drainage catheters in both lungs. C) A computed tomography (CT) scan of the chest on the fourth postoperative day. Decreased mediastinal fluid, decreased pleural effusion on both sides, and atelectasis of the middle lobe were noted (arrow).

**Figure 4:**
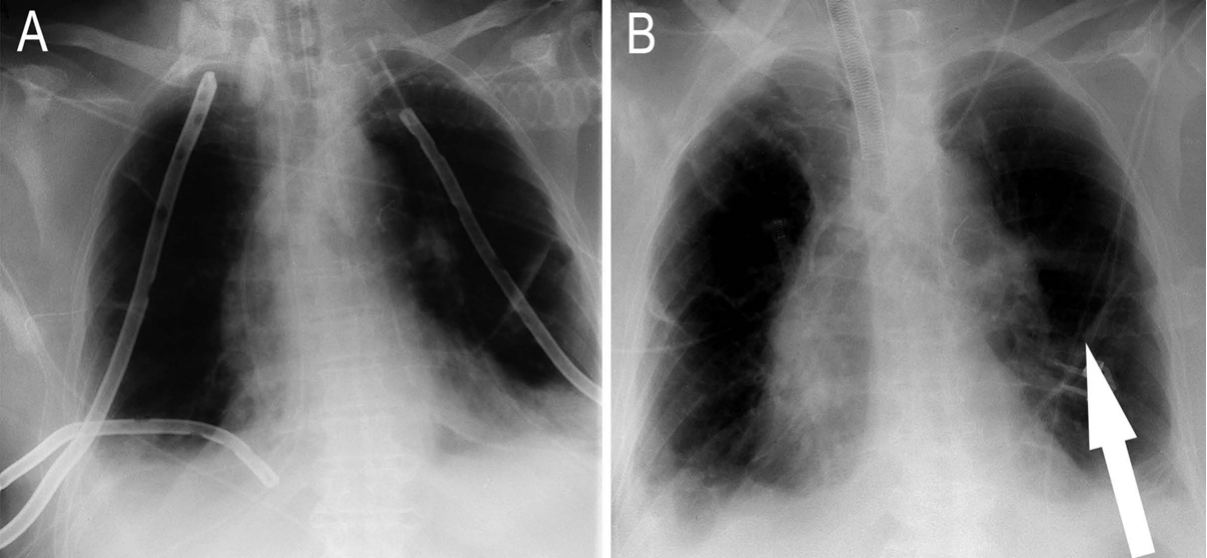
**Follow-up anteroposterior chest radiographs of the chest taken 13 days (A) and 17 days (B) after the first operation**. No pleural effusion and decreasing mediastinal edema with chest tubes in place are visible. Despite improved ventilation of both lungs, basal underventilation with basal striated atelectasis can be observed on both sides and is accentuated on the left side (B). After removal of the chest tubes on both sides, edge shadowing with declining basal underventilation, striated atelectasis (arrows) and a discrete left pleural effusion (B) were found. Note the tracheal cannula inserted after tracheotomy (B).

Revision surgery took place on the second postoperative day, 20 hours after the start of symptoms and 49 hours after the primary surgery. Serous turbid liquid was revealed, with no bleeding and no evidence of esophageal injury. The wound was swabbed, rinsed and drained. Asystole occurred immediately after surgery and was resolved by mechanical and pharmacological resuscitation without further consequences. Our patient's condition was stabilized by volume substitution. A second revision surgery was performed on the third postoperative day. Infection had increased with grey muddy necrosis of the fascia which was excised and sent to our laboratory for histology. After rinsing the wound, a vacuum dressing was applied. Additionally, pleura drainage was performed on both sides (Figure 3A). Our patient was stabilized on catecholamine therapy (epinephrine: 0.16 μg/kg body weight/minute; noradrenaline: 0.112 μg/kg body weight/minute). She was ventilated, intubated and analgosedated, and was transferred to the department of pneumology, thoracic and vascular surgery on the third postoperative evening. The presumptive diagnosis of NSTI of the mediastinum with group A β-hemolytic streptococcus (GAS), with sepsis and empyema of the pleura was verified by the microbiological and histological results over subsequent days. A minithoracotomy with opening of the dorsal and anterior mediastinum and opening of all compartments, necrectomy of the upper paratracheal mediastinum and opening of the pericardiostomy was performed on the fourth post-operative day. A suction lavage drainage catheter was placed in each lung (Figure 3B, C). In addition, a percutaneous endoscopic gastrostomy (PEG) tube was inserted. The antibiotics used were changed to Zienam (imipenem, cilastatin-natrium) and metronidazol. Our patient's condition stabilized remarkably following these interventions (Figure 4).

A further surgical revision, including a change of the vacuum dressing, was performed two weeks later. A circumscribed necrosis of the whole anterior wall of the middle third of the trachea was noted one week later during the dressing change. For that reason, a tracheotomy with a myoplasty of the right sternocleidomastoid muscle was required. The reason for the anisocoria, which had first been observed on the second postoperative day, was glaucoma in our patient's left eye. This was treated without complications in an ophthalmology clinic. After weaning from the respirator, incomplete proximal accentuated tetraparesis in combination with weakness due to inactivity, critical illness myopathy and central apraxia were observed. Our patient was transferred to an early neurological rehabilitation center three months after her initial surgery. At this time, she was tracheotomized but was able to eat and drink independently while the PEG tube remained in place.

**Figure 5:**
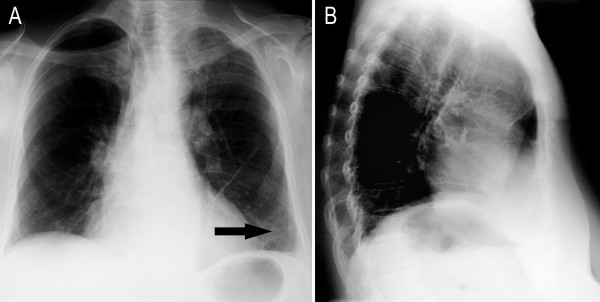
**Chest radiographs in two planes showing a small mediastinum with the pre-existing enlarged cardiac silhouette 19 months after the first operation (compared to Figure 1)**. Both lungs were equally ventilated. Free recess and thin paracardial striated atelectasis with the beginning of a left pleural thickening (arrow) were seen.

Our patient remained immobile and developed a sacral decubitus ulcer, which was treated conservatively. Partial fecal and urinary incontinence were noted. During the three-month rehabilitation period, complete weaning from the respirator, the removal of the tracheal cannula, PEG tube and urinary catheter and mobilization were achieved. Our patient was discharged from hospital six months after her initial surgery. She was continent of feces and urine, was able to attend to her own personal hygiene, could walk 100 m with a walker and was able to climb a flight of stairs (approximately 20 stairs). A further 80 logopedic and 70 ergotherapeutic sessions were necessary to address speaking deficits and loss of motion of her right arm. Our patient had pneumonia of the left upper lobe with cardiac decompensation four months later (16 months after the initial surgery). She was again hospitalized for two weeks and treated with antibiotics for pneumonia. Finally, radiology of the thorax only showed minor residual pathology when compared to the pre-operative X-rays (Figures 1 and 5).

At present, four years after the initial surgery, our patient stated during a telephone evaluation that she has scars which are sensitive to weather changes with the feeling of a ring around her thorax. In addition, she has speaking difficulties under strenuous physical and psychological conditions as well as a loss of strength in her right arm. She lives in her own home, but requires assistance with daily activities and is not employed.

The pre-operative values and their controls until the evening of the first postoperative day were unremarkable. The postoperative laboratory values are reported in Table 1 up to her transfer to the department of pneumology, thoracic and vascular surgery (Table 1). Blood glucose levels were unremarkable. Discrete fluctuations were treated with insulin. The thyroid hormones were normal under daily prophylaxis with 100 μg of thyroxine.

**Table 1:** Postoperative laboratory values reported until transfer of our patient to the center of pneumology, thoracic and vascular surgery.

Parameter	Post-operative day						
	
	First			Second			Third
Time	7 a.m.	8.50 p.m.	11 p.m.	7.30 a.m.	5 p.m.	9.50 p.m.	7 a.m.

CRP (mg/dL)	NA	22.2	NA	30.5	29	NA	38.9

Leukocytes (cells/μL)	9000	6200	4900	3800	7100	13,000	8800

CK (U/L)	NA	177	161	177	127	NA	NA

Creatinine (mg/dL)	0.69	1.81	1,91	1.96	2.21	2.54	2.5

GOT (U/L)	NA	17	17	NA	32	NA	NA

GPT (U/L)	NA	14	13	NA	26	NA	NA

LDH (U/L)	NA	166	168	177	204	NA	NA

Quick (%)	81	NA	61	58	44	43	47

aPTT (seconds)	38.1	NA	44.2	45.6	51.3	59.3	72

AT III (%)	NA	NA	58	NA	NA	54	NA

Thrombocytes (cells/μL)	131,000	133,000	119,000	116,000	104,000	190,000	218,000

aPTT = activated partial thromboplastin time; AT III = anti-thrombin III; CK = creatine kinase; CRP = C-reactive protein; GOT = glutamate-oxaloacetate transferase; GPT = glutamate-pyruvate transaminase; LDH = lactate dehydrogenase; NA = not analyzed.

A nodular colloid goiter with regressive changes was diagnosed in the 9 × 6 × 5 cm right-sided lobulated, resected specimen. There was no evidence of malignancy in the submitted material. The tissue obtained during the second revision surgery showed necrosis and suggested the presence of NSTI. The mediastinal tissue sample taken during the minithoracotomy showed extensive ischemic necrosis with massive bacterial colonization and phlegmonous purulent inflammation. The pericardial biopsy, which was taken during the pericardiostomy in the same operation, showed fibrinous and low phlegmonous granulocytic inflammation.

A lumbar puncture performed on the second postoperative day was sterile. The pleurocentesis revealed serous fluid, which contained leukocytes at a concentration of 20,000 × 10^3 ^cells/μL. No infectious agents were detected in either the immediate swabs or subsequent smears. Microbiological analysis of the intra-operative smear taken during the first revision operation revealed the presence of GAS, which was sensitive to the antibiotics administered. The samples of the pleurocentesis and later samples from the chest tube were sterile. Analysis of the bronchial secretions during the bronchoscopy on the third postoperative day revealed the presence of *Klebsiella oxytoca *and *Candida albicans*. The mediastinal swabs taken during the three revision operations in the thoracic center first showed the previously diagnosed GAS, later *Staphylococcus epidermidis *and *Candida glabrata *and, at the last surgery, *Viridans streptococci*.

The radiological diagnostic tests are shown in figure 1, figure 2, figure 3, figure 4 and figure 5. A cranial computed tomography (CT) scan was performed because of our patient's personality changes. The results of this CT scan were normal. A radiological gastrografin swallow was also performed, which did not show an iatrogenic esophageal lesion. In addition, results of a gastroscopy performed on the third postoperative day were normal. A second cranial CT scan was performed on the fourth postoperative day after the successful resuscitation. No bleeding or infarcts were seen and normal internal and external cerebral fluid spaces were seen. Suspicion of a postischemic lesion in the basal ganglia was raised, but no evidence for this was seen in the following cranial CT.

Negative troponin test results and an electrocardiogram ruled out an acute myocardial infarction on the first postoperative day. Echocardiography after resuscitation did not provide evidence for acute cardiac damage on the second postoperative day. Only a slight impairment of left ventricular systolic function was observed.

Bronchoscopy showed a large volume of turbid purulent secretions on the third postoperative day. Pulmonary function tests showed a forced expiratory volume (FEV1) of 1.1 L, which was 50% of the predicted value, and a vital capacity of 1.7 L (61% of the normal value) 16 months after the recurrent goiter surgery. Blood gas analysis indicated partial oxygen saturation (pO_2_) of 74 mmHg and partial carbon dioxide saturation (pCO_2_) of 41 mmHg.

## Discussion

In principle, NSTI may develop in a completely healthy individual, and the exact cause and focus of the infection often remain unknown [3]. The following risk factors for NSTI have been described in the literature: immunosuppressive therapy such as high-dose corticosteroid therapy, HIV infection, intravenous drug use or substance abuse, individual genetic characteristics, advanced age, previous throat infections, microtrauma of the skin, general trauma, muscle strain, previous surgical interventions and hospitalization in children with *Varicella *infection, black skin color and comorbidities such as diabetes mellitus, alcoholism, hypertension, obesity, peripheral vascular disease, chronic lung disease and tumors [3,4]. In our case obesity, diabetes mellitus, hypertension and tumor nephrectomy with compensated renal insufficiency were identified as risk factors. In the literature, the combination of being aged over 60 years with renal failure and diabetes is associated with a mortality rate of 64.7% [5]. A recent study showed that patients who experienced a craniocervical NSTI with thoracic extension were likely to be older, had more comorbidities, required more surgical debridement, experienced more severe postoperative complications and had a lower overall survival rate of approximately 60% [6].

The initial symptoms of NSTI are highly variable and often nonspecific, which can delay initiation of treatment. This favors a severe course of the disease. Strong diagnostic criteria for NSTI are arterial hypotension or circulatory problems, skin necrosis, bullae and subcutaneous air pockets on radiographs [3]. However, 61% of patients with NSTI do not exhibit these initial symptoms [3]. Pain, which is sometimes difficult to locate and not proportional to the severity of the disease, is reported to be associated with a high mortality in the literature [3,7,8]. In our case, our patient did not report severe pain. The absence of lymphangitis and lymphadenopathy may also be warning symptoms, but were absent in our patient [3]. The skin may initially appear unaffected. A diffuse erythema without defined borders can also occur. The most common initial misdiagnosis is cellulitis in 59% of cases, followed by abscess in 18% of cases, then erysipelas, arthritis, bursitis, deep vein thrombosis and musculoskeletal sprain [3,7]. No effect on the skin was initially observed in our case.

The complications of cervical NSTIs include a high incidence of mediastinal involvement [2]. Jugular vein thrombosis is another commonly associated complication [2]. Our patient had a mediastinitis but no jugular vein thrombosis. Late stages of the disease include disseminated intravascular coagulation, systemic shock and multi-organ failure [7]. In our case, fulminant sepsis developed 29 hours after recurrent goiter surgery with no externally visible skin or wound changes. The first revision surgery was carried out 20 hours after the onset of symptoms. It is likely that the postoperative asystole was caused by a septic toxin inflow. In the literature, the most important reported prognostic factor for patient survival is the time between the onset of clinical symptoms and the first surgical debridement [3].

High values of C-reactive protein (CRP) and creatine kinase (CK) indicate NSTI and, for example, provide evidence against cellulitis [6]. A CT scan can show the extent of the infection, the relationship with contiguous structures, and the necrotic colliquative component [2,3]. CT has proven to be appropriate for use in distinguishing cellulitis (diffuse thickening and infiltration of the cutis and subcutis) from fasciitis (diffuse enhancement and/or thickening of the superficial and deep cervical fasciae) or myositis (enhancement and thickening of the platysma, sternocleidomastoid or strap muscle) [2,9]. Asymmetric thickness of the fascia with fat margins has been found in 80% of cases of NSTI [3]. Routine use of CT is highly recommended in patients with deep cervical infections for early detection of mediastinitis when chest radiography reveals no abnormal findings [2]. In addition, CT provides accurate information on the involvement of the various mediastinal compartments involved in the necrotizing process and is used in determining the optimal thoracic approach for efficient surgical drainage [2].

Mortality is reduced by prompt diagnosis, rapid administration of systemic antibiotics and early surgical debridement [3,4,10]. The aim of therapy should be to perform a surgical revision within 24 hours of symptom onset. This includes smears, collecting specimens for histological analysis and an extensive, aggressive debridement with removal of all necrosis [3]. Intra-operatively, a diminished resistance of the fascia verifiable by the so-called "finger test", a lack of bleeding of the tissue during dissection or a "dishwater" discharge from the wound are often observed [3]. In our case, turbid liquid was found retrosternally in the anterior superior mediastinum. Penicillin and clindamycin represent the gold standard of antibiotic therapy in NSTI of GAS [3,4,10]. Initially, however, *ex juvantibus *broad-spectrum antibiotics are necessary. In our case, piperacillin in combination with gentamicin and vancomycin were given because nosocomial or surgical wound infection could not be excluded. Furthermore, the administration of intravenous immunoglobulin G and activated protein C and hyperbaric oxygen therapy in sepsis are discussed in the literature [3,4,10]. Treatment and monitoring in an intensive care unit are generally required. NSTI may spread rapidly due to the complex fascia system of the neck, including the cervical fascia with the superficial laminae, the prevertebral fascia and the pretracheal fascia. In addition, the rapid spread of infection is facilitated by the peripharyngeal space, which ends caudally in the posterior mediastinum. The descending mediastinitis was diagnosed from CT scans showing increased mediastinal fluid (Figure 2C). Therefore, a minithoracotomy with splitting of the anterior and posterior mediastinal compartment, necrectomy, and the insertion of suction lavage drainage catheters was performed on the fourth postoperative day after goiter surgery. Critical retrospective analysis of the treatment in our case suggests a possible misjudgment (or false estimation) of the first two revision surgeries because only local debridement was performed. A continuous expansion of the infection into the mediastinum had most likely already taken place by the second postoperative day (Figure 2).

In addition, a fibrinous purulent pericarditis was found. Recurrent pericardial effusion with hemodynamic efficacy was suspected. Therefore, the indication for a pericardiostomy was given. To ensure sufficient enteral alimentation in the acute phase of the disease and to avoid the infected area, a PEG tube was inserted. Protracted ventilation with a respirator and a circumscribed necrosis of the trachea in the middle third of the front wall resulting from a distinct NSTI required an open tracheotomy with myoplasty of the right sternocleidomastoid muscle.

## Conclusion

NSTI is a life-threatening disease that can manifest with nonspecific symptoms. Early diagnosis combined with early broad-spectrum antibiotic therapy including a penicillin derivative and prompt radical surgical debridement and observation in an intensive care unit are the essential features of therapy.

## Consent

Written informed consent was obtained from the patient for publication of this case report and the accompanying images. A copy of the written consent is available for review by the Editor-in-Chief of this journal.

## Competing interests

The authors declare that they have no competing interests.

## Authors' contributions

SR and MMB each made substantial contributions to the conception, design, acquisition of data, and analysis and interpretation of data as well as writing the manuscript. All authors read and approved the final manuscript.
